# Reprogramming the immunosuppressive breast cancer microenvironment: integrating cellular, metabolic, and stromal targets for rational immunotherapy

**DOI:** 10.3389/fimmu.2026.1760782

**Published:** 2026-02-13

**Authors:** Chang Ma, Hoon Koon Teoh, Yang Zhao, Yifan Wang, Jun Zhao, Yan Liu, Chen Zhao, Hooi Tin Ong

**Affiliations:** 1 School of Public Health, Jilin Medical University, Jilin, Jilin, China; 2 Department of Preclinical Sciences, M. Kandiah Faculty of Medicine and Health Sciences, Universiti Tunku Abdul Rahman, Kajang, Selangor, Malaysia; 3 Department of Emergency and Critical Care Medicine, No. 965 Hospital of People's Liberation Army Joint Logistics Support Force, Jilin, Jilin, China; 4 Brain Science Institute, Jilin Medical University, Jilin, Jilin, China

**Keywords:** breast cancer, immune evasion, immunotherapy, macrophage plasticity, metabolic reprogramming, tumor microenvironment

## Abstract

Breast cancer, a highly heterogeneous malignancy and a leading cause of cancer-related mortality among women worldwide, is profoundly shaped in its initiation, progression, and therapeutic response by the tumor immune microenvironment (TIME). This review consolidates recent advances in deciphering the cellular, molecular, and metabolic complexity of breast cancer TIME and highlights mechanisms of immune suppression that impede durable treatment efficacy. We critically appraise current and emerging immunotherapeutic approaches, with a focus on strategies that aim to transform immunologically “cold” tumors into “hot,” immune-responsive phenotypes. Novel directions, including metabolic modulation, stromal reprogramming, and precision combination therapies, are discussed in the context of overcoming primary and acquired resistance. We highlight emerging biomarker strategies that integrate spatial transcriptomics to map immune exclusion zones, along with liquid biopsy monitoring of exosomal PD-L1 and circulating tumor DNA, to enable real-time adaptation of combination immunotherapy regimens.

## 1 Introduction

**Figure 1:**
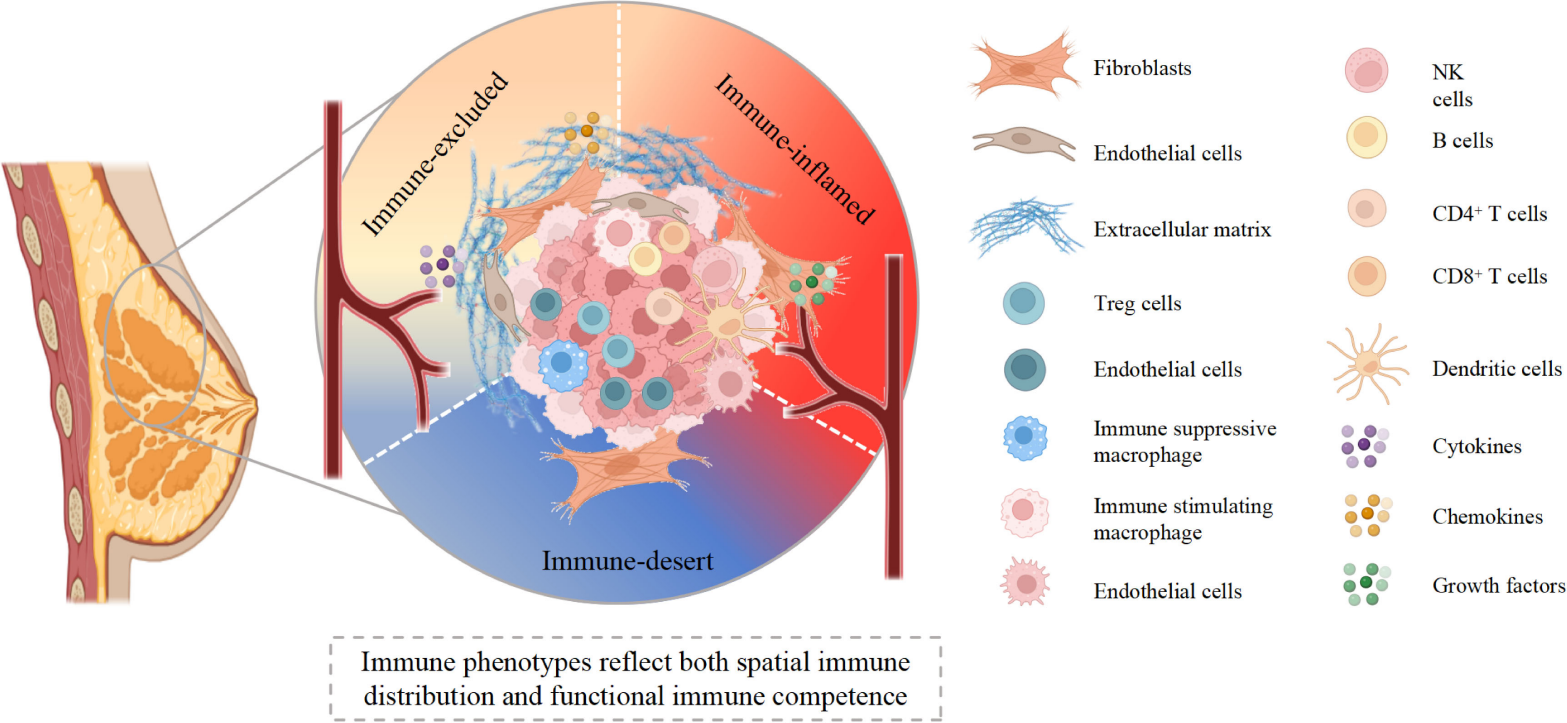
Spatial and functional heterogeneity of the breast cancer tumor microenvironment. This schematic illustrates the continuum of breast tumor immune phenotypes, encompassing immune-desert, immune-excluded, and immune-inflamed states. Immune-desert regions are characterized by minimal lymphocyte infiltration and limited immune activation. Immune-excluded tumors display immune cells retained at the tumor–stroma interface, constrained by dense extracellular matrix, cancer-associated fibroblasts, and aberrant vasculature. Immune-inflamed regions show substantial infiltration of cytotoxic T cells, NK cells, dendritic cells, and B cells, supported by cytokine and chemokine signaling; however, these tumors may still exhibit functionally “cold” features due to impaired T cell priming, immunosuppressive myeloid populations, and T cell exhaustion. Together, spatial immune distribution and functional immune competence jointly define tumor immunogenicity and influence responsiveness to immunotherapy. Created with BioRender.com.

Breast cancer remains one of the most prevalent and biologically diverse malignancies, encompassing distinct subtypes such as hormone receptor-positive (HR^+^), HER2-enriched, and triple-negative breast cancer (TNBC) (1, 2). Despite remarkable therapeutic progress-including advances in surgery, chemotherapy, radiotherapy, and molecularly targeted therapy, a substantial proportion of patients, especially those with metastatic or therapy-resistant disease, continue to experience relapse and poor survival (3, 4). Traditionally, breast cancer research focused on tumor-intrinsic factors such as oncogenic mutations, dysregulated signaling pathways, and proliferative capacity (5). However, an expanding body of evidence now recognizes that tumor behavior and therapeutic response are equally shaped by the surrounding tumor microenvironment (TME) (6, 7). The TME constitutes a complex ecosystem composed of malignant cells, stromal cells (fibroblasts, endothelial cells), immune infiltrates, extracellular matrix (ECM), and soluble mediators such as cytokines, chemokines, and growth factors (8) (**Figure 1**). Within this ecosystem, the tumor immune microenvironment (TIME) plays a decisive role in determining tumor fate and therapeutic efficacy (9, 10). Importantly, the TIME should not be viewed as a binary “immune-cold” or “immune-hot” state, but rather as a dynamic and context-dependent continuum defined by immune composition, spatial distribution, and functional competence. The TIME is not a static entity but a dynamic, continuously evolving interface between cancer and host immunity (11). It includes diverse immune subsets T and B lymphocytes, macrophages, dendritic cells (DCs), natural killer (NK) cells interacting with stromal and vascular components (12, 13) (**Table 1**). These interactions can either support antitumor immunity or reinforce immune evasion (14, 15), depending on their cellular composition, spatial organization (immune-desert, immune-excluded, or immune-inflamed patterns), and spatial organization. In many breast tumors, particularly TNBC, the TIME adopts an immunosuppressive configuration enriched with regulatory T cells (Tregs), myeloid-derived suppressor cells (MDSCs), and M2-polarized tumor-associated macrophages (TAMs) (16).

**Table 1:** Major cellular and molecular components of the breast cancer TIME and their immunomodulatory effects.

Cell type	Key effector molecules / pathways	Immunological function in TIME	Therapeutic target / strategy
CD8^+^ Cytotoxic T Cells	IFN-γ, Granzyme B, Perforin	Direct tumor cell killing; inhibited in “exhausted” state	Immune checkpoint blockade (anti–PD-1/PD-L1, anti–CTLA-4); metabolic reprogramming
CD4^+^ T Helper Cells / Tregs	IL-10, TGF-β, FOXP3	Tregs suppress effector T cells and promote immune tolerance	Treg depletion (anti–CD25, low-dose cyclophosphamide); TGF-β inhibitors
Tumor-Associated Macrophages (TAMs)	CCL18, VEGF, IL-10, Arg1, MMPs	Promote angiogenesis, ECM remodeling, and T-cell anergy	CSF-1R blockade; CD47–SIRPα inhibition; reprogramming via TLR/CD40 agonists
Myeloid-Derived Suppressor Cells (MDSCs)	Arginase-1, ROS, iNOS, TGF-β	Inhibit T-cell proliferation and DC activation; suppress antigen presentation	CXCR2 antagonists; STAT3 inhibitors; FAO blockade (etomoxir)
Dendritic Cells (DCs)	MHC-I/II, CD80/CD86, IL-12	Initiate T-cell priming; often functionally impaired by lactate and TGF-β	STING agonists; FLT3L-DC vaccines; HDAC inhibitors to restore antigen presentation
Cancer-Associated Fibroblasts (CAFs)	CXCL12, IL-6, TGF-β, ECM components (collagen, fibronectin)	Physical barrier to T-cell infiltration; recruit immunosuppressive cells	TGF-β blockade; CXCL12/CXCR4 antagonists; CAF normalization therapy
Endothelial Cells (TECs)	VEGF, PD-L1, FasL, CD39/CD73	Regulate immune cell trafficking; promote “immune exclusion” and adenosine signaling	Vascular normalization (anti-VEGF); CD73/A2AR inhibitors
Natural Killer (NK) Cells	NKG2D, perforin, IFN-γ	Mediate innate tumor cytotoxicity; inhibited by TGF-β and hypoxia	IL-15 agonists; checkpoint inhibitors (anti–NKG2A); adoptive NK therapy
B Cells / Plasma Cells	Antibodies, IL-10, CXCL13	Participate in tertiary lymphoid structures (TLSs); can be regulatory (Bregs)	TLS induction therapies; CD40 agonists; anti-IL-10 strategies
Tumor Cells	PD-L1, IDO1, HIF-1α, CD47	Evade immune detection via checkpoints, metabolic reprogramming, and “don’t-eat-me” signals	PD-L1 blockade; IDO1 inhibitors; metabolic enzyme inhibition (HK2, GLUT1)

Elevated levels of inhibitory cytokines (e.g., IL-10, TGF-β) and immune-checkpoint molecules (e.g., PD-L1) further reinforce immune tolerance (17, 18). Notably, even tumors with substantial immune infiltration may remain functionally immunosuppressed due to defective T cell priming, chronic antigen exposure, and progressive T cell exhaustion, thereby exhibiting “cold” phenotypes despite an immune-inflamed architecture. This milieu contributes to the so-called immune-cold phenotype that underlies poor responsiveness to immune checkpoint blockade (ICB) therapies (19). To unlock the full potential of immunotherapy in breast cancer, it is imperative to understand the mechanisms by which tumor and stromal elements reshape the TIME and subvert immune surveillance (20). Here, we review the cellular and molecular architecture of the TIME in breast cancer; discuss the mechanisms driving immune evasion, including T cell exhaustion, metabolic and epigenetic suppression, and stromal exclusion; highlight therapeutic opportunities to reprogram the TIME through metabolic modulation, re-education of macrophages and fibroblasts, and vascular normalization; and outline future strategies for personalized immunomodulation by integrating spatial multi-omics and artificial intelligence–driven analyses.

## 2 Cellular landscape of the immunosuppressive breast cancer microenvironment

The TIME is orchestrated by a diverse array of immune and stromal populations that collectively determine tumor progression and therapeutic response (21, 22). Within breast cancer, the TIME can either promote antitumor immunity or support tumor immune escape, depending on the relative abundance, spatial organization, and activation states of these cell types.

### 2.1 Lymphocyte populations and functional states

Lymphocytes constitute the adaptive arm of anti-tumor immunity, with their composition, activation state, and spatial distribution fundamentally shaping therapeutic responses in breast cancer. Tumor-infiltrating lymphocytes (TILs) as an emerging immune biomarker are gaining increasing clinical relevance in therapeutic decision-making and the evaluation of immunotherapy trials. Although standardized thresholds defining “high” or “low” TILs have not yet been established, consistent and reproducible assessment of stromal TILs on hematoxylin and eosin (H&E) stained sections is critical for realizing their prognostic and predictive value, particularly in the management of breast cancer (23).

In TNBC, high CD8^+^ TIL density correlates with improved outcomes and immunotherapy sensitivity, whereas ER^+^ tumors typically exhibit sparse lymphocyte infiltration and limited immunogenicity. TILs demonstrate subtype-specific prognostic value. In TNBC, high CD8^+^ TIL density correlates with improved outcomes and immunotherapy sensitivity (22, 24–26), whereas ER^+^ tumors typically exhibit sparse infiltration and limited immunogenicity. Conversely, in ER^+^/HER2^−^ breast cancer, high TILs associate with adverse clinicopathological features, though this relationship is modified by chemotherapy exposure (27). The International Immuno-Oncology Biomarker Working Group on Breast Cancer has developed recommendations for the assessment and reporting of TILs (https://www.tilsinbreastcancer.org/pitfalls/). In future clinical practice, reporting TILs as a potential prognostic and predictive biomarker is becoming increasingly important, and with accumulating evidence, it may play an ever-greater role in the clinical management of individual patients with breast cancer.

#### 2.1.1 CD8^+^ T cell heterogeneity and effector states

**Figure 2:**
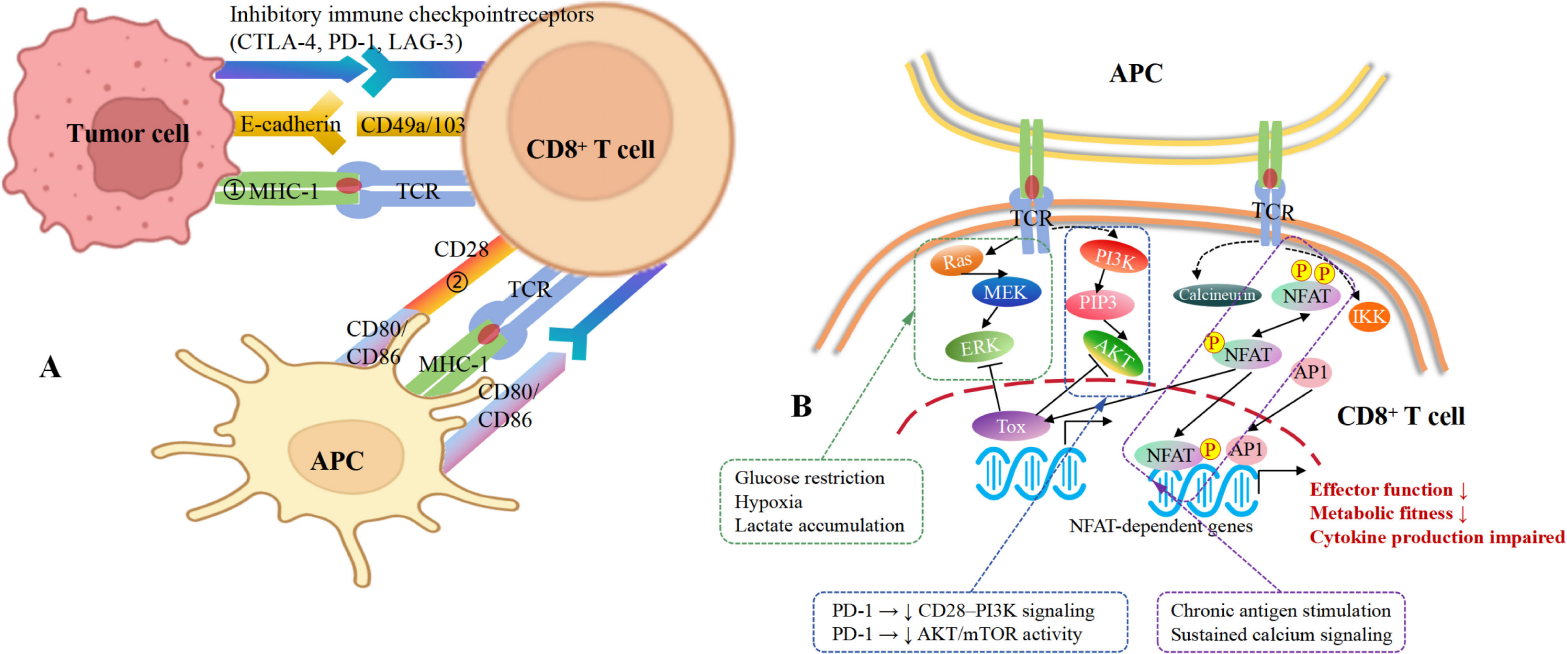
Mechanisms of CD8^+^ T cell dysfunction in breast cancer. **(A)** CD8^+^ T cells interact in many ways with other cells, including tumor cells and APCs. Tumor cells engage T cells through adhesion molecules (E-cadherin, CD49a/103) and present antigen via MHC-I. Concurrently, inhibitory immune checkpoint receptors (CTLA-4, PD-1, LAG-3) expressed on tumor cells or APCs bind their ligands on T cells, while costimulatory molecules (CD80/CD86) interact with CD28, collectively modulating T cell activation, differentiation, and the balance between effector function and suppression. **(B)** Molecular mechanisms underlying intracellular signaling dysregulation that drive CD8^+^ T cell dysfunction and exhaustion. Under conditions of chronic antigen exposure and inadequate costimulation, TCR engagement leads to attenuated activation of key downstream pathways, including MAPK, PI3K–AKT, and IKK signaling, in part due to immune checkpoint-mediated suppression of CD28-dependent signaling. Insufficient activation of these pathways limits nuclear translocation of the transcription factor AP-1. Consequently, sustained NFAT activation in the absence of AP-1 partnership promotes the transcription of anergy- and exhaustion-associated genes, including transcriptional repressors (EGR2, EGR3, IKZF2, IRF4, TOX) and the E3 ubiquitin ligase CBL-B. This transcriptional program establishes a repressive regulatory network that silences effector genes, impairs metabolic and cytokine fitness, and enforces persistent functional unresponsiveness of CD8^+^ T cells within the tumor microenvironment.

CD8^+^ cytotoxic T lymphocytes (CTLs) are central effectors of adaptive anti-improve immunity, yet their functional capacity in breast cancer is highly heterogeneous and context dependent. Although capable of recognizing and eliminating tumor cells, CD8^+^ T cells progressively acquire dysfunctional states during tumorigenesis, contributing to disease progression (28). Early foundational studies established the central role of adaptive and innate immune cells in antitumor immunity well before the advent of modern immunotherapy. Seminal work by Rosenberg and colleagues first demonstrated that tumor-infiltrating CD8^+^ cytotoxic T lymphocytes are capable of mediating tumor regression in solid cancers, laying the conceptual groundwork for T cell–based immunotherapy (29). CD8^+^ T cell dysfunction is not a binary state but rather evolves through distinct, mechanistically defined stages (**Figure 2**). In early-stage breast tumors, CD8^+^ T cells exhibit priming defects characterized by suboptimal costimulation. As tumors progress, persistent antigen exposure combined with an increasingly hostile microenvironment drives these cells toward terminal exhaustion. With tumor progression, persistent antigen exposure, and increasingly immunosuppressive TME drive these cells are driven toward a terminal exhaustion phenotype characterized by sustained expression of multiple inhibitory receptors (e.g., CTLA-4, PD-1, LAG-3), loss of effector functions, and limited reversibility, features reminiscent of exhausted T cells in chronic infection (30). Initial T cell activation requires dual signals: TCR engagement with peptide-MHC complexes (signal 1) and costimulatory interactions (e.g., CD28-CD80/86; signal 2) (**Figure 2A**). In early breast tumors, antigen-presenting cells or tumor cells often lack sufficient costimulatory ligands, resulting in TCR signaling without concomitant costimulation (31). This “signal mismatch” leads to suboptimal activation of downstream pathways, including MAPK, PI3K-AKT, and IκB Kinase (IKK), insufficient to drive robust activator protein 1 (AP-1) nuclear translocation (32). Without AP-1 co-activation, nuclear factor of activated T Cells (NFAT) assumes a “partnerless” conformation and binds regulatory regions of anergy-associated genes (**Figure 2B**). This drives expression of transcriptional repressors (EGR2, EGR3, IKZF2, IRF4, TOX) and the E3 ubiquitin ligase CBL-B, which collectively enforce T cell unresponsiveness by silencing effector programs (33). Together, these factors establish a transcriptional repression network that silences effector genes (e.g., IFNG, TNF) and enforces T cell unresponsiveness. This dysfunction is not a uniform endpoint, but a dynamic program orchestrated by intertwined transcriptional, signaling, epigenetic, and functional perturbations (34).

As tumors advance, chronic antigen stimulation combined with immunosuppressive cues (e.g., TGF-β, IL-10, hypoxia) locks CD8^+^ T cells into an irreversible exhausted state (35). This transition is marked by loss of TCF1 and dominance of TOX-driven exhaustion programs (36). While sharing core features with exhausted T cells in chronic viral infection, tumor-infiltrating CD8^+^ T cells exhibit distinct regulatory nuances shaped by the breast cancer TME. Despite the clinical success of immune checkpoint blockade in some settings, most breast cancer patients show limited response—a challenge rooted in the depth and heterogeneity of T cell dysfunction within the immunoscape.

#### 2.1.2 CD4^+^ T cell subsets: effectors and regulators

CD4^+^ T helper cells orchestrate anti-tumor immunity through cytokine production, B cell activation, and enhancement of CD8^+^ T cell responses. Sauer et al.’s study described the functional specialization of CD4^+^ T helper subsets, emphasizing that Th1-driven cellular immunity is a key determinant of effective anti-tumor responses, while Th2-biased immunity is often associated with tumor progression (37). Lu et al. analyzed 128 breast cancer patients and found that CD57 and PD-1 expression on CD4^+^ T helper cells mark immunosenescence and exhaustion, respectively (38). CD4^+^CD57^+^ T cells are senescent and functionally impaired, with limited proliferation, poor cytokine secretion, and reduced immune activity; their accumulation correlates with systemic immune decline and may promote tumor progression via immunosuppression. CD4^+^PD-1^+^ T cells are exhausted due to chronic antigen exposure, exhibit upregulated PD-1, and fail to recognize or eliminate tumor cells, contributing to immune evasion. Seung et al. demonstrated that CD4^+^ T cells exert antiproliferative effects by arresting tumor cells at the G1/S phase transition (39), with particularly pronounced efficacy in HER2-positive breast cancer. TNF was identified as a critical mediator of this CD4^+^ T cell–dependent growth inhibition, as neutralizing anti-TNF antibodies markedly abrogated their cytotoxic activity, whereas antibodies against IFNγ or IFNα had no such effect.

Tregs, characterized by CD4^+^CD25^+^FOXP3^+^ expression, represent a major immunosuppressive lymphocyte population (40). Tregs suppress effector T cell function through multiple mechanisms. Secretion of inhibitory cytokines (IL-10, TGF-β), competitive consumption of IL-2 via high-affinity CD25 expression, and CTLA-4-mediated downregulation of co-stimulatory molecules on antigen-presenting cells (41). Elevated intratumoral Treg frequencies correlate with poorer outcomes across breast cancer subtypes. The Treg-to-CD8^+^ T cell ratio has emerged as a prognostic biomarker, with high ratios indicating immune suppression dominance. Notably, certain CTLA-4 blocking antibodies selectively deplete intratumoral Tregs through antibody-dependent cellular cytotoxicity (ADCC), contributing to their therapeutic efficacy beyond checkpoint blockade alone (42, 43).

ICB can activate CD4^+^ T cells to produce IL-5 (44). IL-5-producing CD4^+^ T cells and eosinophils synergistically regulate the efficacy of ICB in breast cancer through the IL-5-IL-33 axis. IL-5 drives systemic eosinophil proliferation, IL-33 promotes their invasion into the tumor, and subsequently activates CD8^+^ T cells to enhance anti-tumor immunity. Th1 cells producing IFN-γ and TNF-α promote M1 macrophage polarization and support CTLs’ function, correlating with a favorable prognosis in TNBC.

#### 2.1.3 NK cells and innate lymphoid populations

NK cells serve as pivotal effectors in cancer immune surveillance through natural cytotoxicity and ADCC (45). Early investigations into innate immunity revealed that NKcells exert MHC-independent cytotoxicity against transformed cells and contribute to immune surveillance, particularly in the context of reduced MHC class I expression (46). Their antitumor functions are mediated by the release of lytic granules (e.g., perforin and granzyme B), expression of death receptors (e.g., TRAIL and FasL), and production of cytokines such as IFNγ and TNF-α (47, 48). NK cells engage in bidirectional crosstalk with adaptive immune cells, including conventional T cells and mucosal-associated invariant T (MAIT) cells, enhancing adaptive immunity by promoting DC maturation and recruiting CD8^+^ T cells, while being simultaneously suppressed by Tregs (49). Conversely, adaptive immune cells can also support NK cell maturation and functional maintenance.

NK cells provide MHC-unrestricted innate antitumor surveillance, rendering them an especially attractive therapeutic target in breast cancer, where downregulation of MHC class I molecules is frequent (50). They recognize and lyse tumor cells through activating receptors such as NKG2D, NKp46, and DNAM-1, and exert ADCC via CD16 (49, 51). In breast cancer, NK cell infiltration varies by molecular subtype, with higher frequencies observed in HER2-positive tumors, where trastuzumab-mediated ADCC contributes to improved therapeutic outcomes. However, the tumor microenvironment commonly impairs NK cell function through multiple mechanisms (52). Interactions between NK cells and innate immune populations, including macrophages, MDSCs, DCs, and neutrophils, are similarly bidirectional. NK cell activity can be augmented by activating signals derived from innate immune cells, yet is frequently dampened by inhibitory cytokines or ligands expressed by these same populations (53). Collectively, these dynamic interactions critically shape the immunological architecture of the tumor microenvironment. Strategies to reinvigorate NK cell immunity-including IL-15 superagonists, checkpoint blockade, and chimeric antigen receptor (CAR)-NK cell therapy, are under active investigation in breast cancer. Other innate lymphoid cell (ILC) populations remain poorly characterized in breast tumors (Rethacker et al., n.d.). ILC2s, which produce type 2 cytokines, may promote tumor progression through M2 macrophage polarization, whereas ILC1s producing IFN-γ could support anti-tumor immunity, though their functional significance requires further investigation (54).

#### 2.1.4 B cells and tertiary lymphoid structures

Although historically considered secondary players, B cells were also recognized to influence tumor immunity through antigen presentation and antibody-mediated mechanisms, with both tumor-promoting and tumor-restraining functions described in early breast cancer models (55). B lymphocytes and plasma cells constitute an underappreciated component of the breast cancer immune landscape (56). Tumor-infiltrating B cells organize into tertiary lymphoid structures (TLS), ectopic lymphoid aggregates containing B cell follicles, T cell zones, and high endothelial venules preferentially in immunologically “hot” tumors (57). TLS presence correlates with favorable prognosis and enhanced immunotherapy responses across multiple solid tumors, including breast cancer, likely reflecting organized immune activation and effective antigen presentation (58). In early-stage breast cancer, B-cell infiltration is often associated with a higher rate of pathological complete response. However, during the metastatic phase, B cells may undergo phenotypic or functional transformations such as differentiating into regulatory B cells, shifting from an anti-tumor to a pro-tumor role (59). Infiltration of B cells in the primary tumor is significantly correlated with poor overall survival and a lower probability of radiological complete response in patients.

B cells contribute to anti-tumor immunity through antibody production, antigen presentation to CD4^+^ T cells, and cytokine secretion (60). However, regulatory B cells producing IL-10 can suppress effector T cell responses, adding another layer of immunoregulatory complexity. Recent single-cell studies have revealed functional B cell heterogeneity in breast tumors, with distinct subsets associated with either immune activation or suppression (61). The precise contribution of humoral immunity to breast cancer immunosurveillance remains an emerging area requiring systematic investigation, particularly regarding neoantigen-specific antibody responses and their therapeutic potential (62). Beyond intrinsic dysfunction of T lymphocytes, the immunosuppressive phenotype is further driven by suppressor immune cell subsets.

### 2.2 Myeloid and stromal compartments: key non-lymphoid players

Beyond lymphocytes, breast cancer TIME is profoundly shaped by non-lymphoid cellular populations that collectively enforce an immunosuppressive niche. These include TAMs, MDSCs, cancer-associated fibroblasts (CAFs), and tumor-associated endothelial cells (TECs), each contributing to immune evasion through distinct yet synergistic mechanisms. In line with the recently updated hallmarks of cancer, accumulating evidence further indicates that therapy-induced and stress-associated senescent cells constitute an additional, previously underappreciated, non-lymphoid component of the tumor microenvironment with active roles in disease progression (63).

The myeloid compartment represents a critical axis of immunosuppression in breast cancer, with TAMs constituting up to 50% of the tumor mass in certain subtypes (64). Unlike relatively well-characterized lymphoid populations, myeloid cells exhibit remarkable functional and metabolic plasticity that is exploited by tumors to establish and maintain immune privilege. TAMs are among the most abundant and functionally versatile immune populations within breast TIME. Far from being passive bystanders, TAMs exhibit profound phenotypic and functional plasticity that is dynamically sculpted by tumor-derived signals, therapy-induced stress, metabolic constraints, and spatial niche cues. This plasticity enables TAMs to oscillate between pro-inflammatory (M1-like) and immunosuppressive (M2-like) states or, more accurately, to occupy a spectrum of hybrid and context-dependent activation states that defy binary classification. Additionally, in breast cancer, TAMs are overwhelmingly skewed toward immunosuppressive, pro-angiogenic, and pro-metastatic functions, making them central architects of immune evasion and therapeutic resistance (**Table 2**). Notably, therapeutic interventions such as chemotherapy can further reshape TAM bioenergetics and polarization states, reinforce immunosuppressive programs while simultaneously creating vulnerabilities that may be therapeutically exploited. However, their very plasticity also renders them uniquely targetable, offering a promising avenue for microenvironmental rewiring.

**Table 2:** TAM subpopulations in breast cancer subtypes.

TAM subset	Markers	Metabolic profile	Predominant subtype	Function	Targetability
M1-like	CD80+iNOS+IL-12+	Glycolytic	TNBC (early)	Anti-tumor	Amplify with agonists
M2-like	CD163+CD206+Arg1+	Oxidative	ER^+^, late TNBC	Pro-tumor	CSF1R/CCR2 inhibition
Lipid-laden	PPARγ+FABP4+	FAO-dominant	Obesity-associated	Immunosuppressive	Metabolic inhibitors
SPP1+	SPP1+TREM2+	Mixed	Metastatic	ECM remodeling	Anti-SPP1/TREM2

Historically, TAMs in breast cancer were simplistically categorized as “M2-polarized” based on surface markers (e.g., CD163, CD206) and cytokine profiles (e.g., IL-10, TGF-β). However, scRNA-seq and spatial transcriptomics have revealed a far more complex and heterogeneous landscape. In TNBC, for instance, TAMs co-express both M1- and M2-associated genes, suggesting a hybrid state shaped by conflicting signals from necrotic tumor regions, hypoxic zones, and stromal compartments (65). These “intermediate” TAMs are not transitional-they are stabilized by epigenetic modifications (e.g., H3K27me3 deposition by EZH2) and metabolic reprogramming that constrains nutrient availability and redox balance (e.g., arginase-1 mediated L-arginine depletion), which lock them into a pro-tumorigenic identity resistant to conventional immunomodulation (66). Similarly, lipid-laden TAMs in obese or high-fat diet-associated breast cancers exhibit PPARγ/δ-driven oxidative metabolism that sustains immunosuppressive cytokine production and limits effective antigen presentation and T cell support (64). Moreover, emerging evidence suggests that senescent stromal and myeloid cells within breast cancer TME further amplify these immunosuppressive circuits. Once considered predominantly tumor-suppressive, senescent cells are now recognized as active drivers of tumor progression through the senescence-associated secretory phenotype (SASP), which comprises pro-inflammatory cytokines, chemokines, growth factors, and matrix-remodeling enzymes (63). SASP factors can reinforce TAM recruitment and polarization, enhance CAF activation, promote angiogenesis, and blunt anti-tumor immune surveillance, thereby reshaping the microenvironment toward a chronically inflamed yet immunologically permissive state. These findings underscore that TAM polarization is not solely cytokine-driven but is metabolically hardwired, a paradigm shift with profound therapeutic implications.

### 2.3 Cancer-associated fibroblasts: architects of the immunosuppressive stroma

Beyond immune cells, pioneering studies identified as active regulators of tumor progression rather than passive structural components. Orimo et al. first demonstrated that CAFs promote breast tumor growth and angiogenesis through paracrine signaling, thereby indirectly shaping immune cell recruitment and function within the tumor microenvironment (67). CAFs represent a numerically dominant and phenotypically diverse stromal population, often exceeding tumor cell abundance in desmoplastic breast cancers. Once considered passive structural scaffolds, CAFs are now recognized as active orchestrators of immunosuppression, ECM remodeling, and therapeutic resistance (68). In breast cancer, CAFs arise from multiple cellular origins-including resident fibroblasts, mesenchymal stem cells, adipocytes, and even endothelial or epithelial cells via epithelial-mesenchymal transition (EMT), contributing to their phenotypic and functional diversity (69, 70).

Recent scRNA-seq studies have revealed distinct CAF subpopulations in breast cancer, notably iCAFs, myCAFs, and apCAFs, each exhibiting unique secretomes and immunomodulatory capacities (71). While myCAFs, characterized by high α-smooth muscle actin (α-SMA) expression, drive ECM stiffening and physical barriers to T cell infiltration, iCAFs secrete cytokines such as IL-6, CXCL12, and TGF-β that suppress CTL function and promote regulatory Treg expansion (72). Notably, apCAFs - expressing MHC class II molecules but lacking co-stimulatory signals - may induce T cell anergy rather than activation, thereby subverting adaptive immunity (73). The spatial organization of CAFs within the TME dictates their immunosuppressive impact. Peritumoral CAFs often form dense, collagen-rich “exclusion zones” that physically impede T cell trafficking into tumor nests - a hallmark of immune-cold breast cancers, particularly in TNBC and HER2-negative subtypes (71). Moreover, CAF-derived TGF-β not only induces fibroblast-to-myofibroblast differentiation but also directly inhibits CD8^+^ T cell effector function and promotes Treg differentiation, creating a self-reinforcing immunosuppressive loop (74).

### 2.4 Endothelial cells and the network of intercellular crosstalk

Tumor-associated endothelial cells (TECs) are not merely passive conduits for blood flow but dynamic regulators of immune cell trafficking, angiocrine signaling, and immunosuppression within the breast TME. In breast cancer, abnormal tumor vasculature-characterized by disorganized architecture, leakiness, and hypoxia-creates a physical and biochemical barrier to effective immune surveillance (75). This aberrant endothelium expresses reduced levels of adhesion molecules (e.g., ICAM-1, VCAM-1) and chemokines (e.g., CXCL9/10), impairing leukocyte adhesion and transendothelial migration, thereby contributing to the “immune-excluded” phenotype observed in many breast tumors (76, 77).

Beyond structural dysfunction, TECs actively participate in immunosuppressive crosstalk. They upregulate immune checkpoint ligands such as PD-L1 and FasL, directly inducing T cell apoptosis or exhaustion (78). Moreover, TECs secrete soluble mediators, including VEGF, endothelin-1, and TGF-β, that inhibit DC maturation and promote Treg and MDSC recruitment (79). Recent spatial proteomics studies in TNBC have revealed endothelial niches enriched in CD39^+^/CD73^+^ ectonucleotidases, which catalyze extracellular ATP into immunosuppressive adenosine, further dampening T cell responses (80).

The reciprocal interactions between TECs and stromal constituents represent a mechanistically complex yet frequently overlooked determinant of the TME. Cancer-associated fibroblasts secrete VEGF and IL-6, which potentiate endothelial barrier dysfunction and upregulate PD-L1 expression on TECs. Conversely, endothelial-derived Notch ligands promote fibroblast activation, thereby establishing a positive feedback circuit that perpetuates immunosuppressive microenvironmental conditions (81). Furthermore, TECs conditioned by tumor signals can phenotypically reprogram macrophages toward an alternatively activated (M2-like) state through exosomal microRNA-105 transfer and additional paracrine mediators, underscoring the integrated architecture of stromal communication networks (82).

From a therapeutic perspective, vascular normalization, defined as the functional restoration rather than ablation of tumor vasculature, has garnered considerable interest in facilitating immune cell trafficking. Intermittent, low-dose administration of anti-angiogenic agents such as bevacizumab transiently restructures aberrant tumor vessels, resulting in improved perfusion, alleviation of hypoxia, and enhanced CD8^+^ T cell infiltration (83). In preclinical breast cancer models, particularly TNBC, the combination of vascular normalization with ICB has demonstrated synergistic antitumor efficacy (83). Nonetheless, the therapeutic interval for this approach remains constrained; excessive VEGF inhibition precipitates vascular regression and exacerbates hypoxia, thereby attenuating the immunotherapeutic response.

Advancing this field necessitates a refined understanding of endothelial heterogeneity within the tumor vasculature. Single-cell RNA sequencing has delineated functionally distinct TEC subpopulations in breast cancer, including tip cells, stalk cells, and interferon-responsive endothelial cells (IRECs), the latter potentially facilitating lymphocyte extravasation during inflammatory conditions (84). Selective targeting of specific endothelial subsets or their intercellular signaling axes, such as the angiopoietin-2 (ANG2)/TIE2 and delta-like ligand 4 (DLL4)/Notch pathways, may afford precision modulation of vascular-immune crosstalk while minimizing off-target toxicities (85).

## 3 Immunosuppressive mechanisms and drug resistance pathways in breast cancer

The TME in breast cancer functions as a highly organized and adaptive ecosystem that not only enables immune evasion but actively drives resistance to both conventional therapies and contemporary immunotherapeutic approaches (86). Achieving durable clinical responses, therefore, requires systematic dismantling of this immunosuppressive niche. While immune checkpoint blockade-particularly targeting the PD-1/PD-L1 axis-has demonstrated clinical benefit in a subset of patients, its limited efficacy underscores the need to elucidate additional resistance mechanisms (87). We focus on three interconnected and mechanistically distinct paradigms. First, metabolic rewiring, wherein tumor cells reprogram local metabolism to monopolize critical nutrients and generate immunosuppressive metabolites that impair immune cell function; Second, non-canonical immune checkpoints and T cell exhaustion signatures, which are upregulated as compensatory pathways following PD-1/PD-L1 inhibition and contribute to T cell dysfunction; and last the immunosuppressive stroma, which establishes both a physical barrier to immune infiltration and a signaling hub that sustains an anti-inflammatory, pro-tumorigenic milieu. Importantly, these mechanisms converge on myeloid cells, positioning them as key integrators of therapeutic resistance and rational targets for combination strategies with immune checkpoint inhibitors. By synthesizing these advances, we provide a cohesive and forward-looking framework for the development of next-generation combinatorial strategies aimed at reprogramming immunologically “cold” breast tumors into “hot,” immune-responsive phenotypes.

### 3.1 Metabolic reprogramming: the immunosuppressive fuel switch

Tumor-driven metabolic reprogramming constitutes a mechanistically distinct but clinically underexplored axis of immunosuppression in the breast cancer TME stems from tumor-driven metabolic reprogramming. Tumor-driven metabolic reprogramming creates competitive nutrient depletion hostile to immune effectors. Heightened aerobic glycolysis (Warburg effect), driven by PI3K/AKT/mTOR and HIF-1α, monopolizes glucose required for TIL activation and cytotoxic function (88, 89). Concurrently, aggressive consumption of glutamine and tryptophan generates immunosuppressive metabolites: IDO1-mediated tryptophan catabolism produces kynurenine, which inhibits effector T cells while promoting Treg differentiation (90). The metabolic shift generates byproducts that are intrinsically immunosuppressive. The massive production of lactate from accelerated glycolysis is a key resistance mechanism. High lactate concentrations lead to the acidification of the TME, which significantly impairs the lytic activity of CTLs and NK cells (60). Moreover, lactate acts as a signaling molecule: it promotes the polarization of TAMs towards the pro-tumoral M2 phenotype and enhances the suppressive function of MDSCs (91).

Beyond impairing cytotoxic lymphocyte function, lactate serves as a potent immunomodulatory signal that actively reprograms myeloid cells. In breast cancer, tumor-derived lactate stabilizes HIF-1α in TAMs through direct inhibition of prolyl hydroxylases (PHDs), the oxygen-sensing enzymes responsible for HIF-1α degradation. Simultaneously, lactate serves as a substrate for histone lysine lactylation, a recently identified post-translational modification wherein lactyl-CoA, derived from glycolysis-generated lactate, is transferred to lysine residues on histone tails by p300 acetyltransferase. This epigenetic mark at gene promoters such as Arg1 and VEGF maintains their transcriptional activation independent of continuous cytokine signaling, effectively “locking” TAMs into an immunosuppressive, M2-like phenotype (92, 93) (**Table 3**). Notably, chemotherapeutic pressure can further accentuate these metabolic adaptations, reshaping TAM bioenergetics and reinforcing polarization states that dampen antigen presentation and T cell priming unless actively counteracted by myeloid-targeting interventions. Cassetta et al. further describe how chemotherapy-induced reprogramming of circulating monocytes and TAMs in breast cancer leads to aggressive disease progression, with gene signatures linked to poor outcomes, and uncover signaling loops involving SIGLEC1, CCL8, and CSF1 that may serve as potential biomarkers and therapeutic targets for modulating the tumor immune microenvironment (94).

**Table 3:** Metabolic targets in breast cancer immunotherapy.

Pathway	Key enzyme/molecule	Inhibitor	Mechanism	Clinical status	Combination partner
Glycolysis	LDHA	FX11, Oxamate	↓Lactate production	Preclinical	Anti-PD-1
Tryptophan catabolism	IDO1	Epacadostat	↓Kynurenine	Phase III (failed), reassessing	Anti-PD-1
Adenosine	CD73	Oleclumab	↓Adenosine	Phase II	Anti-PD-L1
Adenosine	A2AR	Ciforadenant	Block suppressive signaling	Phase I/II	Anti-PD-1
Glutamine	GLS1	CB-839	↓Glutamine metabolism	Phase II	Various

Importantly, metabolic reprogramming within breast cancer TME is not limited to enhanced glycolysis but also involves a dynamic shift toward mitochondrial oxidative phosphorylation (OXPHOS), particularly within immune and stromal compartments (95). In effector CD8^+^ T cells, sustained antitumor activity requires mitochondrial fitness and OXPHOS-dependent bioenergetics to support memory formation and long-term persistence (96). Conversely, immunosuppressive populations, including Tregs and M2-like TAMs, preferentially rely on fatty acid oxidation–driven OXPHOS, a metabolic state that supports suppressive function, longevity, and resistance to nutrient deprivation. Tumor- and therapy-induced cues can therefore promote an OXPHOS-dominant metabolic program in myeloid cells, reinforcing immune tolerance while limiting effective T cell responses (97). This context-dependent mitochondrial switch underscores the need for carefully timed metabolic interventions, as indiscriminate inhibition of OXPHOS may impair protective immunity alongside suppressive programs.

**Figure 3:**
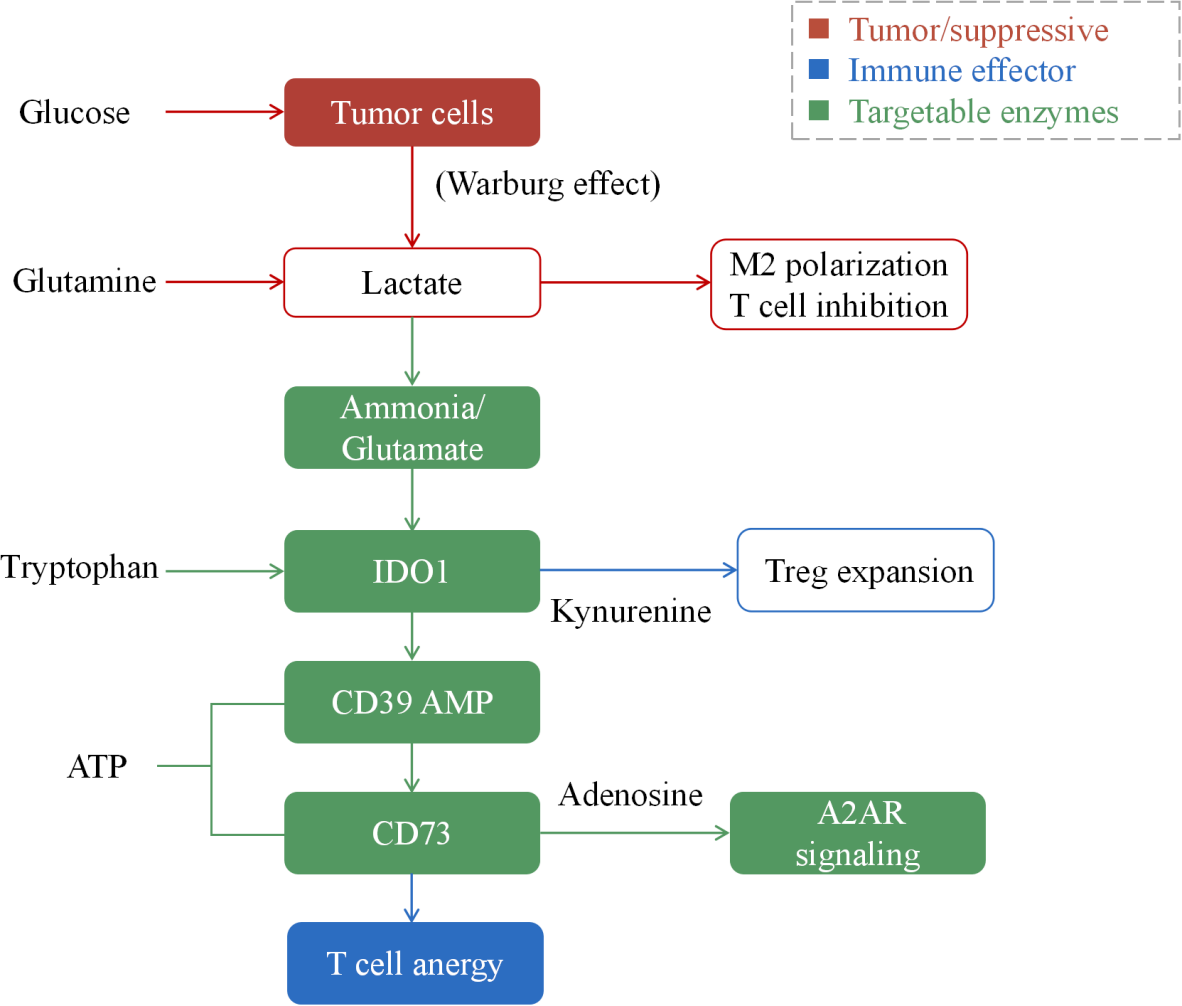
Tumor cells (red) drive immunosuppression through metabolic reprogramming. Aerobic glycolysis (Warburg effect) consumes glucose, generating lactate, which inhibits T cell function and promotes M2 macrophage polarization. Concurrently, tryptophan catabolism via IDO1 produces kynurenine, expanding regulatory T cells (Tregs). The purinergic pathway, initiated by ATP release and hydrolyzed by CD39/CD73 (green), generates adenosine, which signals through A2AR to induce T cell anergy (blue). These interconnected pathways create a nutrient-depleted, metabolically hostile microenvironment that actively suppresses anti-tumor immunity.

An emerging and highly targetable immunosuppressive pathway involves the purinergic signaling cascade, often termed the “adenosine checkpoint” (98). Hypoxia and high cellular turnover lead to the release of ATP into the extracellular space. This ATP is sequentially hydrolyzed by the ectonucleotidases CD39 and CD73 (Ecto-5’-nucleotidase), which are frequently overexpressed on breast cancer cells, CAFs, and Tregs (**Figure 3**). The final product, adenosine, binds to A2A and A2B receptors (A2AR/A2BR) on effector immune cells. This signaling cascade dramatically increases intracellular cAMP, resulting in the inhibition of T cell proliferation, cytokine production (e.g., IFN-γ), and cytotoxicity (99). This pathway not only enforces immune tolerance but also limits the efficacy of immune checkpoint blockade, providing a strong rationale for combinatorial targeting.

The metabolic vulnerability inherent in the breast cancer TME offers a potent, yet complex, avenue for next-generation immunotherapeutic design (100). The primary challenge in leveraging this vulnerability lies in achieving a high degree of selective metabolic targeting, disrupting the hyper-glycolytic and immunosuppressive pathways of tumor and stromal cells while preserving mitochondrial programs required for effective and durable T cell immunity (101). Combination strategies that integrate myeloid-targeting agents with immune checkpoint inhibitors are emerging as a promising approach. By reprogramming TAM metabolism and function, such combinations can relieve myeloid-driven immune suppression, enhance antigen presentation, and synergize with therapies that induce regulated cell death, thereby promoting durable T cell-mediated immunity rather than transient tumor control (99, 102).

Ultimately, the successful clinical implementation of these strategies requires high-resolution spatial and functional mapping. Advances in technologies like single-cell metabolomics and spatial transcriptomics are indispensable for precisely delineating the distinct metabolic states of heterogeneous tumor, stromal, and immune cell subpopulations, thereby enabling the rational selection of targets for precise reversal of the immunosuppressive fuel switch (103–105).

### 3.2 Non-canonical immune checkpoints and exhaustion signatures

While the clinical success of ICB targeting the PD-1/PD-L1 and CTLA-4 axes has been transformative, resistance in breast cancer, especially in the highly immunogenic TNBC subtype, often involves the compensatory activation of a repertoire of non-canonical inhibitory receptors (106, 107). These emerging checkpoints serve as critical gatekeepers, driving profound T cell dysfunction and establishing a state of sustained immune exhaustion.

High-dimensional analyses have consistently identified the functional upregulation and co-expression of next-generation checkpoints, including TIM-3, LAG-3, and TIGIT, on TILs. LAG-3 binds to MHC Class II and Fibrinogen-like protein 1 (FGL1), directly inhibiting TCR signaling and antigen presentation efficacy (108). TIM-3 is often co-expressed with PD-1 on severely exhausted T cells, signaling via its ligands (e.g., galectin-9) to mediate T cell apoptosis and suppress IFN-γ production (109, 110). TIGIT competes with the activating receptor CD226 for binding to CD155 (PVR) and CD112 (PVRL2), thereby suppressing cytotoxic function and promoting the immunosuppressive capacity of regulatory T cells (111, 112). These receptors do not operate in isolation; rather, their concurrent expression acts synergistically to elevate the inhibitory threshold of T cells. The activation of these pathways is frequently observed upon ICB failure, suggesting they serve as intrinsic resistance mechanisms by sustaining T cell exhaustion when the PD-1 pathway is neutralized.

A major breakthrough facilitated by scRNA-seq has been the realization that T cell exhaustion is not a uniform endpoint but a spectrum of distinct cellular states with varying responsiveness to therapy (113). This phenotypic heterogeneity is defined by the unique patterns of inhibitory receptor co-expression and transcriptional profiles. Within the exhausted T cell compartment, two functionally and transcriptionally distinct subsets have been consistently delineated across multiple solid tumors, including breast cancer: progenitor exhausted and terminally exhausted T cells. Progenitor exhausted T cells are defined by intermediate expression of PD-1 and sustained expression of the transcription factor TCF1 (encoded by TCF7), which confers stem-like properties such as self-renewal and proliferative potential. This subset retains metabolic flexibility and epigenetic accessibility, enabling robust proliferative responses to PD-1 blockade; upon PD-1/PD-L1 inhibition, progenitor exhausted T cells can undergo robust clonal expansion and differentiate into effector-like states, thereby serving as the principal mediators of therapeutic response to ICB monotherapy (114). This dichotomy carries profound therapeutic implications. In breast cancer patients receiving PD-1 blockade, clinical responders demonstrate higher baseline frequencies of TCF1^+^ progenitor exhausted cells, whereas non-responders are enriched for TCF1^-^ terminally exhausted populations (115). Furthermore, the progenitor-to-terminal ratio may serve as a dynamic biomarker: early on-treatment expansion of progenitor cells correlates with durable responses, while their rapid depletion predicts primary resistance (116). These findings underscore the need for combination strategies that either preserve progenitor cells (metabolic rescue) or reprogram terminal cells (epigenetic modulators) to overcome exhaustion-mediated resistance.

In contrast, terminally exhausted T cells exhibit a fixed dysfunctional phenotype characterized by the co-expression of multiple inhibitory receptors, including PD-1, TIM-3, LAG-3, and the complete loss of TCF7. These cells display profound and largely irreversible impairments in cytokine production, cytotoxicity, and proliferative capacity. Their persistence in the TME is often contingent upon survival signals derived from stromal or myeloid compartments, and they demonstrate minimal responsiveness to current ICB monotherapies (117). The progenitor-terminal divide has direct therapeutic implications, as ICB efficacy depends on preserving or expanding the progenitor exhausted pool while developing strategies to reprogram or bypass terminal exhaustion, key challenges in advancing immunotherapy for immunologically “cold” or resistant breast cancer subtypes.

### 3.3 The stroma as a physical and molecular resistance shield

The stromal compartment, primarily composed of CAFs and the dense ECM, functions as a formidable and dynamic barrier that actively promotes therapeutic resistance in breast cancer (118). CAFs, which are often the most abundant non-malignant cell type, orchestrate a complex immunosuppressive environment that transcends simple physical containment.

Recent advances, largely driven by single-cell transcriptomics, have dissolved the concept of CAFs as a monolithic entity, revealing a highly heterogeneous population with distinct molecular signatures and functional roles (e.g., myofibroblastic, inflammatory, or senescent CAF subsets) (119, 120). These subpopulations drive immune evasion through both biomechanical and biochemical means. The stiff, collagen-rich ECM remodeled by CAFs creates a physical barrier that impedes the effective migration and infiltration of CTLs into the tumor core.

Biochemically, CAFs are potent secretors of chemokines and cytokines that actively promote immune exclusion. A prime example is the production of C-X-C Motif Chemokine Ligand 12 (CXCL12), which binds to its receptor CXCR4 on T cells (121).

High peritumoral CXCL12 gradients establish a “chemokine trap” that redirects CXCR4^+^T cells away from tumor nests through competing migratory cues. Mechanistically, prolonged CXCR4 engagement triggers receptor internalization and desensitization via β-arrestin recruitment, rendering T cells unresponsive to tumor-derived chemokines such as CXCL9/10. Additionally, CXCL12 activates PI3K/AKT signaling in T cells, paradoxically promoting their survival in stromal zones while maintaining them in a quiescent, non-cytotoxic state. This traps T cells outside the tumor parenchyma and prevents their necessary interaction with cancer cells, a mechanism highly associated with poor outcomes and resistance to ICB (122). Furthermore, specific CAF subsets secrete immunosuppressive factors such as IDO1 or TGF-β, which enhance the recruitment of MDSCs and Tregs, shifting the overall immune profile towards tolerance (123).

The influence of CAFs extends beyond immune suppression to the direct induction of resistance to non-immunological therapies, highlighting a mechanism of cross-resistance that complicates combination regimens. CAF-secreted factors, such as fibroblast growth factor 2 (FGF2) or certain chemokines, can activate pro-survival signaling pathways (e.g., PI3K/AKT/ERK) in cancer cells (124). This activation can render ER^+^ breast cancer cells resistant to endocrine agents or targeted therapies, and critically, may also contribute to the tumor’s resistance to T cell-mediated killing (125). CAFs shield tumor cells from chemotherapy through multiple mechanisms by promoting cancer stem cell (CSC) plasticity or mediating drug efflux (126).

Given their central role, CAFs represent a highly logical yet challenging therapeutic target. Non-selective ablation of CAFs has been largely unsuccessful in clinical settings due to unintended pro-tumorigenic effects mediated by specific subsets (119). Therefore, the future lies in precision-targeting specific immunosuppressive CAF subpopulations (e.g., inflammatory or senescent CAFs) or neutralizing their key resistance pathways. Targeting the CXCL12/CXCR4 axis with antagonists, in combination with ICB, holds immense promise for disrupting T cell sequestration, enhancing TIL infiltration, and remodeling the fibrotic “cold” TME into an immune-receptive one (121). Moreover, identifying and neutralizing the CAF-derived factors (e.g., FGF2, specific chemokines) that drive metabolic or survival cross-resistance offers a necessary strategy to restore sensitivity to both targeted and immune-based therapies simultaneously (125). This intricate approach requires advanced spatial technologies to precisely map CAF subsets and their associated molecular resistance mechanisms within the physical constraints of the tumor microenvironment.

### 3.4 Emerging resistance mediators: exosomes and microbial influences

A truly innovative and rapidly expanding domain in resistance research focuses on non-cellular and exogenous mechanisms that exert profound, often systemic, influence over anti-tumor immunity. These factors include extracellular vesicles (Exosomes) and the recently discovered intratumoral microbiome (127).

#### 3.4.1 Exosomes as nanoscale propagators of immunosuppression

Tumor-derived exosomes (TDEs) are nanosized extracellular vesicles that function as crucial mediators of intercellular communication within the TME and at distant metastatic sites (128). TDEs carry a biologically active cargo-including proteins, oncogenic mRNAs, and immunosuppressive microRNAs, which they rapidly transfer to recipient immune cells, endothelial cells, and stromal components (129, 130). This transfer constitutes a highly efficient, systemic mechanism for propagating the immunosuppressive phenotype.

TDEs exert immunosuppression through multiple cargo types. Surface-displayed PD-L1 engages PD-1 on cytotoxic T cells, functioning as circulating checkpoint molecules that systemically suppress T cell activation (129, 131). Exosomal microRNAs, particularly miR-21 and miR-155, target TLR8 signaling in dendritic cells, impairing their capacity for antigen presentation and T cell priming (132). In HER2^+^ breast cancer, TDEs sequester trastuzumab through surface-bound HER2, reducing effective drug concentration (35).

Importantly, accumulating evidence indicates that chemotherapy itself can reprogram the exosomal landscape in breast cancer, thereby promoting metastatic dissemination. Chemotherapy-induced exosomes have been shown to remodel the tumor microenvironment by enhancing pro-metastatic signaling, including Annexin A6–dependent membrane dynamics and CCL2-mediated recruitment of inflammatory monocytes, ultimately facilitating metastatic niche formation and tumor cell extravasation (133). These findings highlight a paradoxical role of cytotoxic therapy in amplifying EV-mediated communication that favors tumor spread and immune evasion.

Furthermore, TDEs can reprogram macrophages towards the immunosuppressive M2 phenotype and suppress the lytic function of NK cells, contributing to resistance against ICB and targeted therapies (134). TDEs also condition distant organs for metastatic colonization by transferring factors that induce vascular permeability and recruit immunosuppressive cells.

In HER2-positive breast cancer, TDEs have been found to carry active HER2 and other proteins that can bind and sequester targeted therapeutic antibodies like Trastuzumab, reducing drug concentration at the primary tumor site and providing an additional mechanism of resistance (135). This illustrates a sophisticated mechanism where exosomes act not just as signaling vehicles but as molecular shields for the primary tumor.

While the role of TDEs in immune resistance is established, several critical challenges and opportunities remain. Phenotypic specificity, a major frontier, lies in distinguishing the functional heterogeneity of exosomes based on the breast cancer subtype. TDEs from TNBC are likely to carry distinct cargo compared to those from ER^+^/HER2^-^ tumors, reflecting differences in their primary resistance mechanisms (e.g., higher enrichment of lytic factors like FasL in TNBC-TDEs to induce T cell apoptosis) (136). Detailed single-exosome analysis is required to define these subtype-specific “immunosuppressive signatures.” From a translational perspective, the abundance of immune-modulatory cargos such as exosomal PD-L1 in circulation positions TDEs as promising liquid biopsy biomarkers to predict therapeutic response and monitor resistance to immunotherapy (137). Therapeutically, future strategies may involve developing exosome-tethered inhibitors or agents that block the biogenesis or uptake of immunosuppressive TDEs, effectively disarming the tumor’s systemic communication network and synergizing with existing immunotherapies.

#### 3.4.2 The intratumoral microbiome: a novel immunomodulator

An entirely novel and high-impact concept is the functional role of the intratumoral microbiome (ITM) - the presence of specific bacterial taxa residing within the tumor tissue itself (138). Although research in breast cancer is nascent, recent data from other solid tumors and preliminary breast cancer studies suggest that the composition and load of ITM can significantly influence local immune responses and chemotherapy efficacy (139).

The ITM drives resistance and modulates the immune TME through several distinct, yet interconnected, mechanisms. First, the ITM can trigger the activation of chronic inflammatory signaling. Specific intratumoral bacteria, such as species of Fusobacterium or Mycoplasma, can activate TLRs on resident tumor cells or immune cells (e.g., macrophages) (140). This activation leads to the chronic release of pro-inflammatory cytokines, notably IL-6 and IL-1 β, which orchestrates a state of sterile, non-resolving inflammation. Crucially, this persistent inflammatory signaling fosters the recruitment and differentiation of highly immunosuppressive cells, including MDSCs and Tregs, thereby effectively transforming the TME into a highly refractory, “cold” environment despite the presence of inflammatory markers. Second, the modulation of local metabolites by the ITM acts as a potent, yet insufficiently explored, immunosuppressive lever (141). Intratumoral bacteria metabolize local nutrients, generating compounds that directly inhibit T cell function. For example, some bacteria can produce high levels of IDO inducing metabolites or compounds that significantly alter the local pH, creating an acidic and nutrient-deprived environment highly unfavorable for effector T cell survival and cytotoxic function (142). Finally, the ITM can influence adaptive immunity through the mechanism of molecular mimicry and cross-reactivity (143). Bacterial antigens may share epitopes with tumor-associated antigens (TAAs). In some scenarios, this cross-reactivity may backfire, promoting the generation of regulatory T cells that recognize both microbial and tumor antigens, thus enhancing systemic immune tolerance and tumor evasion. Conversely, in a paradoxical, beneficial mechanism, this molecular mimicry can sometimes drive the activation of cross-reactive CD8^+^ T cells through what is termed “Bystander T cell Activation”, where T cells recognizing the microbial epitope are activated and subsequently attack the shared tumor epitope (144, 145).

The recognition of exosomes and the ITM as functional, non-cellular, and exogenous mediators of immune resistance opens two highly innovative and intertwined research frontiers that aim to systematically overcome the myriad resistance pathways established by the advanced TME. The first frontier involves exosome-based diagnostics and therapeutics: Tumor-derived exosomes (TDEs) carry a high-fidelity molecular signature of the parent tumor, making them exceptional candidates for liquid biopsy biomarkers to non-invasively monitor dynamic changes in PD-L1 status, predict individual patient response to ICB, or identify the emergence of resistance pathways (146). Therapeutically, this involves developing strategies to disarm the tumor’s systemic communication network, either by inhibiting exosome biogenesis or uptake, or by utilizing engineered exosomes as highly biocompatible carriers for targeted drug delivery to immune or stromal cells (147, 148). The second frontier centers on modulating the immunomicrobiome axis: The ITM and the broader gut microbiome present a compelling target for therapeutic intervention, offering the possibility of converting resistance-driving microbial profiles into immune-enhancing ones (149). Future strategies will explore tailored antibiotic regimens to selectively deplete immunosuppressive intratumoral taxa, or the use of specific probiotics or fecal microbiota transplantation (FMT) to therapeutically rewire the ITM/immune axis to synergize with immunotherapy (149). Ultimately, leveraging these novel mediators offers powerful opportunities to enhance patient stratification, personalized treatment, and design sophisticated combination therapies that simultaneously target both the cellular and non-cellular components driving resistance (150).

## 4 Future perspectives and challenges

The meticulous dissection of the breast cancer immunoscape has unveiled a complex, multifaceted ecosystem, offering unprecedented opportunities to re-engineer the TME for enhanced therapeutic efficacy. While ICB has transformed the management of a subset of breast cancers, particularly TNBC, significant challenges remain, primarily concerning primary and acquired resistance in many patients and less immunogenic subtypes like HR^+^/HER2^−^ disease. The future of breast cancer immunotherapy hinges on moving beyond PD-1/PD-L1 monotherapy towards a deeper, more actionable understanding of TME biology.

### 4.1 Decoding spatial heterogeneity and dynamic immune states

Current analytical approaches, such as bulk and single-cell sequencing, provide invaluable molecular snapshots of the TME but critically fail to capture the essential spatial relationships between cellular architects (e.g., Cancer-associated fibroblasts, endothelial cells) and immune effectors. To fully understand the stroma as a physical and molecular barrier, future advancements must prioritize high-multiplex spatial transcriptomics and proteomics (e.g., GeoMx, CODEX) to precisely map the exact locations of immune exclusion zones, immunosuppressive niches, and active immune synapses in both pre-treatment and on-treatment biopsies. This spatial resolution is paramount for designing strategies that effectively dismantle these localized resistance shields (151). While spatial technologies provide unprecedented resolution of the tissue-level immune architecture, they are limited by sampling bias and invasiveness, capturing only snapshots of a single tumor region at discrete timepoints. To complement this, real-time monitoring of systemic immune dynamics is essential. Furthermore, the TME is an inherently dynamic entity, constantly being reshaped by therapy and tumor evolution, highlighting a major clinical challenge: the lack of robust, non-invasive biomarkers for real-time monitoring of immune responses and the prediction of acquired resistance. Overcoming this requires integrating liquid biopsies (analyzing circulating tumor DNA, circulating immune cells, and exosomes) with advanced machine learning models trained on vast, longitudinal spatial and single-cell datasets. This integrated approach is essential to transition breast cancer treatment from static, one-size-fits-all regimens to adaptive, response-driven therapeutic strategies (152).

The TME is a highly dynamic entity, perpetually reshaped by therapeutic intervention and the relentless process of tumor evolution, underscoring the critical challenge of the lack of robust, non-invasive methods to monitor immune responses and predict acquired resistance in real-time. To overcome this, the future of oncology lies in integrating liquid biopsies, which capture the systemic molecular landscape through circulating tumor DNA (ctDNA), circulating immune cells, and immunosuppressive exosomes with cutting-edge machine learning (ML) models (153). These ML models must be trained on comprehensive, multi-dimensional, and longitudinal data that incorporates both bulk molecular features and spatially-resolved single-cell profiling from pre-treatment and on-treatment biopsies (154). The integration of these modalities, specifically, utilizing the dynamics of ctDNA clearance or exosomal PD-L1 levels from liquid biopsy alongside the initial spatial immune context of the TME, allows for the development of adaptive, response-driven therapeutic strategies (155). For instance, a high-performing ML model integrating RNA, clinical features, and digital pathology has already shown high accuracy in predicting neoadjuvant treatment response in breast cancer, moving treatment away from static, one-size-fits-all regimens toward a true precision oncology framework (156–158). Longitudinal monitoring, particularly of ctDNA dynamics, has been shown to predict disease progression significantly earlier than conventional radiographic response, proving its value as a real-time surveillance tool for resistance mechanisms in patients receiving ICB (159, 160).

### 4.2 Emerging Frontiers in Breast Cancer Immunotherapy: From TME Reprogramming to Personalized Cellular Therapeutics

The future of breast cancer immunotherapy is defined by a strategic evolution focused on expanding the therapeutic repertoire and aggressively pursuing personalized technological frontiers. Beyond the established PD-1/CTLA-4 axes, therapeutic advancement requires validating non-canonical immune checkpoints (LAG3, TIGIT, TIM3) and, crucially, addressing the root cause of T cell anergy: metabolic exhaustion. This is achieved by targeting the immunosuppressive “fuel switch” within the TME, which is driven by competition for essential nutrients. Strategies involve small molecules that inhibit key metabolic enzymes (e.g., IDO1, A2AR, Glutaminase) to restore T cell fitness, particularly when combined with ICB (88, 98, 142, 161). Representative rational combinations currently under clinical evaluation are summarized in **Table 4**. Among these combinations, early clinical data suggest distinct efficacy patterns by breast cancer subtype. In TNBC, the combination of pembrolizumab with nab-paclitaxel (KEYNOTE-355) achieved significant progression-free survival benefit in PD-L1^+^ tumors, while the addition of A2AR antagonists (AZD4635) to anti-PD-L1 therapy is under investigation to overcome adenosine-mediated resistance. In ER^+^ disease, combining CDK4/6 inhibitors with immunotherapy (e.g., abemaciclib plus pembrolizumab) is being tested based on preclinical evidence that CDK4/6 inhibition enhances antigen presentation and reduces Treg suppression. A parallel paradigm shift necessitates moving beyond lymphocyte-centric approaches to actively reprogram the immunosuppressive non-immune compartments. This includes myeloid reprogramming aimed at shifting TAM from a pro-tumoral M2-like state to an anti-tumoral M1-like state, and CAF normalization, which utilizes agents that interfere with TGF-β signaling or mechanosensing to revert CAF to a permissive phenotype without detrimental depletion (68, 71, 120). Simultaneously, technological frontiers are focused on precision, leveraging the neoadjuvant setting as an *in vivo* laboratory for subtype-specific responses: this entails optimizing ICB combinations with PARP inhibitors or T cell engagers for TNBC, while exploring combinations with CDK4/6 or PI3K inhibitors-known for their immunomodulatory effects to convert HR^+^/HER2^–^ “cold” tumors into “hot” ones (73, 165). Finally, next-generation cellular and vaccine therapies are on the horizon, including advancements in CAR-NK cells or TCR-engineered T cells designed to overcome TME suppression and antigen escape. Most transformative are personalized mRNA or dendritic cell vaccines, tailored to the patient’s unique neoantigen landscape, which hold the potential to elicit a robust, *in situ* immune response that fundamentally reshapes the tumor immunoscape (160, 166).

**Table 4:** Rational combination strategies in clinical development.

Combination	Mechanistic rationale	Breast cancer subtype	Clinical phase	Key references / Trial ID
Anti-PD-1 + CD73 inhibitor	Block adenosine generation from extracellular ATP; reverses T/NK cell suppression	TNBC	Phase II	98; NCT03454451
ICB + TGFβ trap (e.g., Fresolimumab)	Disrupt CAF-mediated T cell exclusion and ECM deposition; promotes TIL infiltration	TNBC, HR^+^	Phase I/II	162
Anti-TIGIT + anti-PD-L1	Target co-expressed checkpoints on exhausted CD8^+^ T cells; enhance reinvigoration	TNBC	Phase III	112; SKYSCRAPER-01 (NCT04294810)
Anti-PD-L1 + PARP inhibitor (e.g., Olaparib)	Induce immunogenic cell death + STING activation in BRCA-mutant tumors; synergize with ICB	TNBC (gBRCA^+^)	Phase III	163; KEYLYNK-009
Anti-PD-1 + Anti-angiogenic (e.g., Bevacizumab)	Normalize tumor vasculature → improve T cell trafficking + reduce hypoxia-driven immunosuppression	TNBC	Phase II	83; NCT02734160
Anti-LAG-3 + anti-PD-1	Target terminally exhausted T cells co-expressing LAG-3/PD-1; restore effector function	TNBC	Phase II	108; NCT04859984
ICB + IDO1 inhibitor (e.g., Epacadostat)	Prevent tryptophan catabolism → reduce kynurenine → reverse Treg induction & T cell anergy	HR^+^/HER2^-^, TNBC	Phase I/II (mixed results)	90; ECHO-204 (NCT03329846)
Anti-PD-1 + CXCR4 antagonist (e.g., BL-8040)	Block CXCL12-mediated T cell sequestration in stroma; enable TIL penetration into tumor nests	TNBC	Phase II	121; COMBAT/KEYNOTE-146
Trastuzumab + anti-PD-1	HER2-targeted ADCC + checkpoint blockade enhances CD8^+^/NK activity; dual innate/adaptive activation	HER2^+^	Phase II	164; PANACEA (NCT02129556)
CAR-T/NK + TGFβR inhibitor	Protect adoptively transferred cells from TGFβ-mediated suppression in stroma-rich TME	TNBC (preclinical/early clinical)	Phase I	161; NCT05239143

### 4.3 Challenges and future considerations for breast cancer immunotherapy

Translating these mechanistic insights into clinical benefit requires addressing several implementation challenges. Foremost is defining the “immunological vulnerability” of individual tumors, the rate-limiting factor (e.g., T cell exclusion vs. metabolic suppression vs. checkpoint dominance) that dictates combination therapy selection. Not all components of the TME contribute equally to immunosuppression, so the challenge lies in precisely identifying the rate-limiting immunological factor for a given patient and tumor subtype. This precision is essential to ensure that combination therapies are rational and not merely additive in toxicity.

Furthermore, the inevitable increase in combination immunotherapies raises significant concerns about compounded immune-related adverse events (irAEs). Future research must, therefore, focus on developing targeted delivery systems or novel agents that maximize local immune stimulation within the TME while minimizing systemic toxicity. Another critical consideration is the heterogeneity of metastatic sites. The immune landscape can vary significantly between the primary tumor and metastatic lesions. Therapeutic strategies must account for this organ-specific heterogeneity, necessitating the sampling and analysis of multiple metastatic sites to guide truly personalized treatment (167).

From a translational and implementation standpoint, it is also important to recognize that while cutting-edge spatial transcriptomic and multi-omic platforms have greatly advanced our understanding of the TME, their widespread clinical adoption remains constrained by cost, tissue requirements, and regulatory limitations. In this context, multiplex immune-histochemistry and cyclic immunofluorescence-based approaches represent a highly relevant and accessible alternative for spatially resolved immune profiling (168). These technologies enable simultaneous detection of multiple, clinically validated biomarkers on formalin-fixed paraffin-embedded specimens, allowing robust neighborhood and cell-cell interaction analyses within the TME (169). Importantly, such approaches are compatible with current CLIA-certified workflows and leverage well-established antibody panels, thereby serving as a practical bridge between pathologists, clinicians, and researchers for integrative spatial immune analysis, particularly in resource-limited settings (170).

In conclusion, rewiring the breast cancer immunoscape demands a concerted effort to integrate high-resolution omics with functional assays and spatial analysis. Strategic use of scalable spatial profiling tools, alongside advanced multi-omic technologies, will be essential to translate immune ecosystem insights into clinically actionable biomarkers and therapeutic decisions. The shift from broadly targeted checkpoint blockade to precision immune modulation of all TME components, lymphocytes, myeloid cells, and stroma will define the next era of successful breast cancer immunotherapy, ultimately converting resistant and immune-cold tumors into treatable, immune-responsive diseases.
